# Synergistic Antitumor Effects of Berbamine and Paclitaxel through ROS/Akt Pathway in Glioma Cells

**DOI:** 10.1155/2017/8152526

**Published:** 2017-08-13

**Authors:** Feng Jia, Shu Ruan, Ning Liu, Linshan Fu

**Affiliations:** ^1^Department of Neurosurgery, The First Affiliated Hospital with Nanjing Medical University, Nanjing, China; ^2^Department of Neurosurgery, Yancheng City No. 1 People's Hospital, The Fourth Affiliated Hospital of Nantong Medical College, Yancheng, China; ^3^Department of Endocrinology, Yancheng Third Hospital, The Affiliated Hospital of Southeast University Medical College, Yancheng, China

## Abstract

In our preliminary study, Berbamine (BA), one of the most commonly used traditional Chinese medicines, was effective in inducing the intracellular ROS levels. Since the regulation of cellular antioxidant capacity is crucial to the sensitivity of Ptx, it is feasible to propose that sensitizing cells to Ptx can be achieved through increasing the antioxidant capacity by codelivering BA. Cytotoxicity test demonstrates that either single or combinational treatment of BA and Ptx dose-dependently inhibits the proliferation of U-87 cells. Median-effect analysis clearly proves the synergistic anticancer effect between BA and Ptx. Combinational treatment of both drugs induced more intracellular ROS generation than either of the drugs did. Cotreatment of NAC could partially reverse the ROS generation and ameliorate the cytotoxicity induced by BA plus Ptx. Moreover, sequential activation of ROS-dependent phosphor-Akt expression was dose-dependently inhibited by the combinational application of BA and Ptx, which was more significantly effective than the single treatment of either BA or Ptx. Additionally, the coadministration of BA and Ptx shows the strongest tumor delaying effect in a U87 xenograft model, demonstrating the synergism between two drugs. Therefore, BA is a promising adjuvant to traditional chemotherapy, especially in combination with Ptx, to treat malignant glioma.

## 1. Introduction

Malignant glioma is one of the leading causes of cancer-related mortality for patients who suffer from brain tumor all over the word [1]. Overall survival of glioma has extended because of early detection and comprehensive therapeutic regimens including operation, radiotherapy, and chemotherapy. The prognosis of malignant glioma has not been improved substantially yet due to the lack of effective adjuvant therapy with lower toxicity [2].

Paclitaxel (Ptx) has been approved by FDA to treat a series of solid tumors including breast, ovarian, gastric, and non-small cell lung cancer [3, 4]. As reported previously, Ptx exerts its antitumor effect through interfering with normal mitotic function by stabilizing the microtubule assembly from tubulin and preventing the depolymerization, thereby finally leading to a G2/M cell cycle arrest [5]. Moreover, there are increasing studies demonstrating the advantage of Ptx in combination with other anticancer agents in treatment of malignant glioma [6, 7].

However, its clinical application is severely restricted by both the high incidence of hypersensitivity and the emerging resistance, which often leads to the failure of chemotherapy [8, 9]. A lot of groups have done extensive studies to try to elucidate the possible mechanisms of multidrug resistance [10]. As reported in earlier studies, the cytotoxicity of Ptx depends on the redox state of cells and resistance to Ptx is proportional to cellular total antioxidant capacity [11, 12]. Since the regulation of cellular antioxidant capacity is very crucial to the sensitivity of Ptx, it is feasible to sensitize cells to Ptx through increasing the antioxidant capacity by codelivering preoxidants.

Berbamine (BA), one of the most commonly used traditional Chinese medicines, is a small molecule compound extracted from* Berberis amurensis* (xiaoboan). It has been used for treating hypertension and deficiency of white blood cells for a long time [13, 14]. As reported previously, BA possesses potential antitumor effects against different cancer cell lines including glioblastoma [15, 16]. Moreover, it is also reported that BA could reverse the multidrug resistance [17]. Additionally, BA could effectively restore the sensitivity of several kinds of cancer cells to chemotherapy and radiation therapy [18].

In the current study, BA was chosen as a preoxidant to synergize with Ptx. The synergistic antitumor effect of BA and Ptx was evaluated in both in vitro and in vivo tumor models. A series of cellular and molecular biological methods were utilized to elucidate the possible mechanisms underlying the synergy.

## 2. Materials and Methods

### 2.1. Reagents

Ptx, N-acetylcysteine (NAC), dimethyl sulfoxide (DMSO), 2,7-dichlorodihydrofluorescein diacetate (H_2_DCF-DA), and 3-(4,5-dimethylthiazol-2-yl)-2,5-diphenylformazan (MTT) were purchased from Sigma Chemical Co. (St. Louis, MO, USA). Ptx was dissolved in DMSO. H_2_DCF-DA and NAC were dissolved in PBS. The solutions were then filtered through a 0.22 *μ*m filter (Immobilon, Millipore Corp., Bedford, MA, USA) with further dilutions before use. BA was purchased from Zhejiang Jinhua CONBA Bio-pharm, Co. (Jinhua, China). Cultured medium (RPMI-1640), fetal bovine serum (FBS), and penicillin/streptomycin were obtained from Gibco BRL (Grand Island, NY, USA). Annexin V-fluorescein isothiocyanate (FITC) kit was purchased from Bender MedSystems (Vienna, Austria). Other reagents of analytic grade were obtained from Nanjing Chemical Reagent Co. (Nanjing, China). Human malignant glioma cancer cell line U87 was obtained from the Institute of Biochemistry and Cell Biology, Chinese Academy of Sciences (Shanghai, China).

### 2.2. Methyl Thiazolyl Tetrazolium (MTT) Assay

5000 U87 cells were seeded per well in 96-well plates. Cells were then treated with different concentrations of Ptx (8, 16, 32, 64, and 128 ng/mL), BA (2, 4, 8, 16, and 32 *μ*g/mL), or both drugs (Ptx: 0.25, 0.5, 0.8, 1, 1.2, 1.6, and 2 ng/mL; BA: 0.25, 0.5, 1, 1.5, 2, 3, and 4 *μ*g/mL) in serum-free conditions for 24, 36, or 48 h. In another experiment, cells were pretreated with NAC at 400 *μ*M for 2 h and then treated singly or in combination with different concentrations of Ptx (2, 4, 8, 16, and 32 ng/mL) or/and BA (0.5, 1, 2, 4, and 8 *μ*g/mL) for 48 h. Untreated cells in serum-free medium were used as controls. Cell viability was measured by MTT assay. The interaction of the two drugs was quantified by determining the combination index (CI) according to the method as described previously [19].

### 2.3. DAPI Staining and Flow Cytometry

U87 cells were seeded in 6-well plate containing glass cover slips and allowed to grow for at least 24 h. Cells were then treated with Ptx (1 ng/mL), BA (1.5 *μ*g/mL), or both drugs for 48 h. In another experiment, the cells were pretreated with NAC at 400 *μ*M for 2 h and then treated with Ptx or/and BA. U87 cells were washed once with PBS and fixed with 500 *μ*l fixing solution (acetic acid : methanol; 1 : 3) for 5 min, dried, and stained with the DNA-specific fluorochrome DAPI (1.5 *μ*g/ml). After incubation in the dark at 37°C for 15 min, the cells were washed with PBS, air-dried, mounted with 90% (v/v) glycerol, and examined using a fluorescence microscope (Olympus, Japan). Apoptotic cells were identified by condensation and fragmentation of nuclei. To quantify the apoptosis, cells were treated with Ptx (1 ng/mL), BA (1.5 *μ*g/mL), or both drugs for 48 h and then stained with annexin V-FITC kit (eBioscience, Inc., San Diego, CA, USA) by FACS.

### 2.4. Detection of Intracellular ROS Production

H_2_DCF-DA was chosen as a ROS indicator. 1 × 10^5^ U87 cells were seeded per well in 6-well plates and allowed to grow for 24 h. The cells were then treated with H_2_O_2_ (100 *μ*M, positive control), Ptx (1 ng/mL), BA (1.5 *μ*g/mL), or a combination of Ptx and BA for 6 h. Cells were incubated with 10 *μ*M H_2_DCF-DA for 30 min in dark and fixed with 4% paraformaldehyde. In another experiment, cells were pretreated with NAC at 400 *μ*M for 2 h and then treated with Ptx or/and BA. The fluorescent images were viewed by a fluorescence microscopy (IX-71; Olympus, Tokyo, Japan). The fluorescent intensity was then measured by a fluorescent plate reader (CytoFluor II, Applied Biosystems, Foster City, CA, USA) with excitation and emission wavelengths of 485 and 530 nm, respectively.

### 2.5. Western-Blot Analysis

U87 cells were seeded in 60 mm dishes and exposed to Ptx (1 ng/mL), BA (1.5 *μ*g/mL), or both drugs for 6 h. In another experiment, the cells were pretreated with NAC (400 *μ*M) for 2 h and then treated with or without Ptx or/and BA. Total proteins were extracted by RIPA kit and the proteins were run on a SDS gel for electrophoresis. After being transferred to a PVDF membrane, the proteins were incubated with different primary antibodies including rabbit anti-p-Akt (Ser 473) and anti-Akt (CST, Danvers, MA, USA) and mouse anti-*β*-actin (Sigma, St. Louis, MO, USA). After being washed by PBS for 3 times, the membrane was incubated with goat anti-rabbit and anti-mouse HRP-conjugated secondary antibodies.

### 2.6. In Vivo Study

Male nude mice of 6–8 weeks old were purchased from the animal center of Nanjing Medical University. The in vivo experiments were in compliance with guidelines approved by the Animal Care Committee of Nanjing Medical University. First, 50 *μ*L PBS suspension containing 5*∗*10^6^ U87 cells was injected at the right hind limb of nude mice. Treatment started on Day 0 when the tumors reached 80–100 mm^3^. The mice were randomized to the following four groups: saline group; Ptx alone (10 mg/kg); BA alone (30 mg/kg); and combination of BA (30 mg/kg) and Ptx (10 mg/kg). The drugs were injected once on Day 0. Tumor nodules were measured every other day during the treatment. The volume of tumor was calculated according to the following equation (*W*^2^*∗L*)/2. *W* represents the measurement at the widest point and *L* represents the dimension at the longest point.

### 2.7. Statistical Analysis

Statistical analyses were made by SPSS (version 11.0; SPSS Inc., Chicago, IL, USA). Data were shown as mean ± SD. Significance was analyzed either by Student's *t*-test or one-way ANOVA. A probability value of less than 0.05 was accepted as statistically significant.

## 3. Results

### 3.1. Effects of Ptx and BA on the Proliferation of U-87 Cells

**Figure 1:**
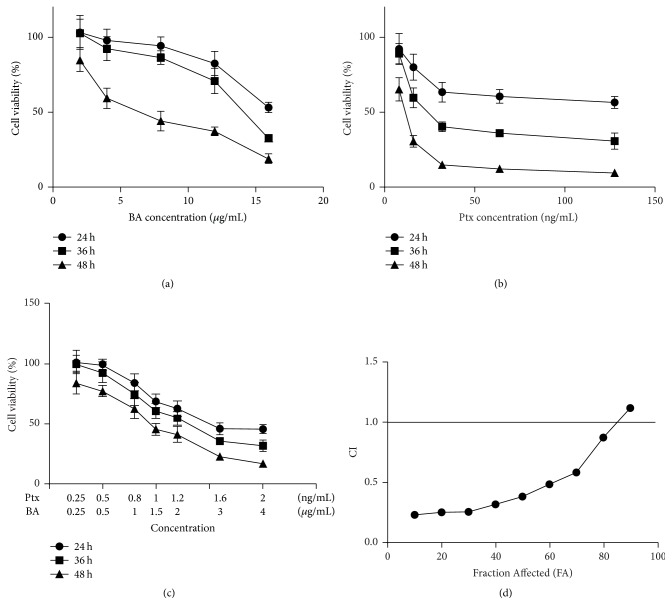
Analysis of synergy between BA and Ptx on U-87 cells. (a) Time and dose-response cytotoxicity curves of BA on U-87 cells. (b) Time and dose-response cytotoxicity curves of Ptx on U-87 cells. (c) Time and combinational dose-response cytotoxicity curves of BA and Ptx on U-87 cells. (d) CI values at different level of growth inhibition effect (Fraction Affected, FA) for 48 h incubation. Values represent mean ± SD (*n* = 3).

It is indicated in Figures 1(a) and 1(b) that BA and Ptx inhibited cell growth with a dose dependent manner against U-87 cells. The IC50 values of BA or Ptx decrease with the extension of incubation time (Table 1).  Figure 1 illustrates the multiple drug effects obtained from U-87 cells which were treated simultaneously with BA and Ptx and represented as fractional cell growth inhibition (FA) as a function of the CI. It is noticed that drug combination in a lower dose generated similar cell death as either of the drugs in higher doses (Figure 1(c)). For instance, a nontoxic dose of Ptx (2.34 nM) and BA (6.42 *μ*M) caused significantly cell death when cells were exposed to the drug combination for 24 or 48 h. The CI analysis (Figure 1(d)) based on the results of 48 h cytotoxicity indicated that CI values were below 1 when the two drugs were combined for 48 h incubation. Media-effect analysis demonstrated a synergistic anticancer effect of BA and Ptx against U-87 cells. The calculated IC50s (Table 1) of the two drugs in single or in combination confirmed the synergistic antitumor effect of BA on Ptx. Either Ptx or BA in combination showed a significantly lower IC50 dose than in single use at each time.

### 3.2. NAC Partially Blocks the Antitumor Effects of Ptx and BA

**Figure 2:**
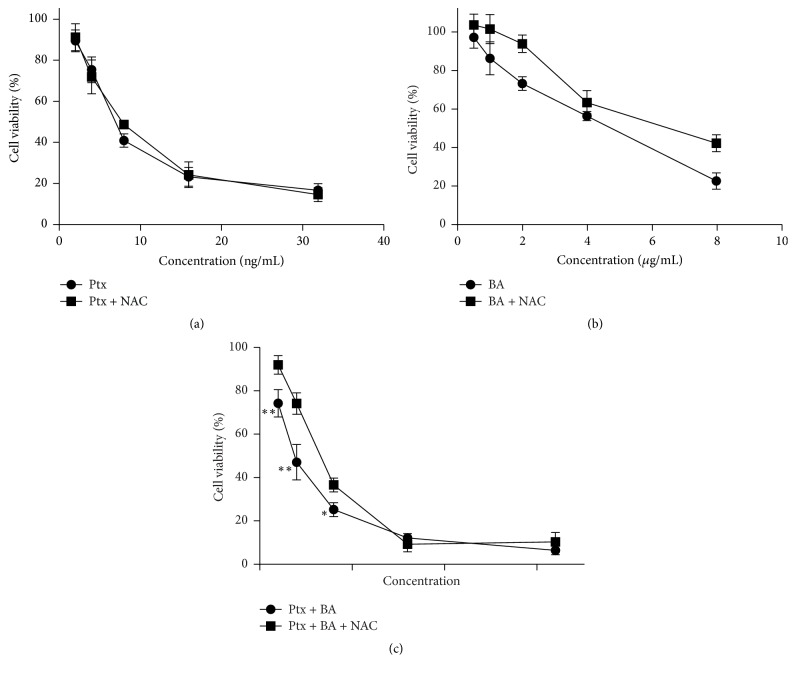
Influence of NAC pretreatment on the cytotoxicity of different agents. (a) The viability of cells exposed to a series of doses of BA after NAC (400 *μ*M) pretreatment. (b) The viability of cells exposed to a series of doses of Ptx after NAC (400 *μ*M) pretreatment. (c) The viability of cells exposed to BA plus Ptx after NAC (400 *μ*M) pretreatment. Values represent mean ± SD (*n* = 3). *∗* means *p* < 0.05 versus equivalent dose group without NAC pretreatment. *∗∗* refers to *p* < 0.01 versus equivalent dose group without NAC treatment.

In order to determine whether ROS generation is involved in Ptx and BA induced proliferation inhibition and apoptosis induction, we evaluated the modulation of antiproliferative and apoptotic effect of Ptx and BA by NAC. As shown in Figures 2(a) and 2(b), pretreatment of NAC showed no obvious influences on the cytotoxicity of Ptx while it did show some protective effect on cells treated with BA. On the contrary, NAC pretreatment significantly increased cellular viability when U-87 cells were exposed to a series of doses of BA and Ptx simultaneously (Figure 2(c)).

### 3.3. Effects of Ptx and BA on the Apoptosis of U-87 Cells

**Figure 3:**
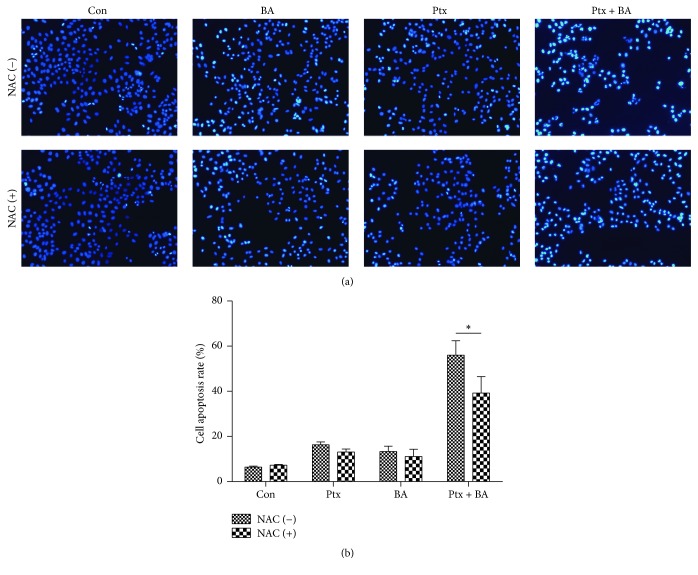
Apoptotic induction of BA and Ptx in single use or combination. (a) Apoptosis of U-87 cells detected by DAPI staining. (b) Quantitative analysis of apoptotic rate of cells exposed to different agents. Values represent mean ± SD (*n* = 3). *∗* means *p* < 0.05 versus the corresponding group.

**Figure 4:**
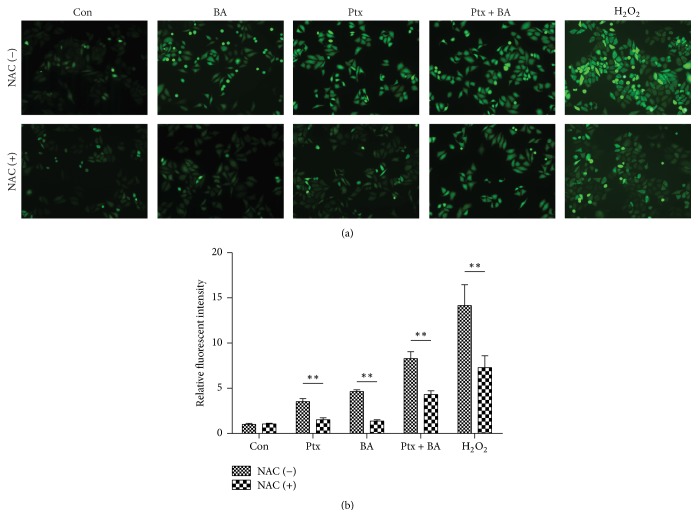
Intracellular ROS levels induced by different agents and the influence of NAC on the induction. (a) Fluorescent pictures of cells exposed to either single or combinational treatment of BA and Ptx with or without the pretreatment of NAC. H_2_O_2_ was applied as a positive control. (b) Quantitative intracellular DCF fluorescence intensity of U-87 cells treated with different agents. All of the values were from the images recorded by fluorescent microscopic images. Values represent mean ± SD (*n* = 3). *∗∗* represents *p* < 0.01 versus the corresponding group.

Apoptosis is initially characterized by morphological features, such as chromatin condensation, nuclear fragmentation, and membrane blubbing. The characteristic morphological changes of apoptosis such as condensation of chromatin and nuclear fragmentation were clearly observed by DAPI staining (Figure 3). To further quantify the number of apoptotic cells treated with Ptx and BA, flow cytometry analysis was performed. As shown in Figure 4, both Ptx and BA induced apoptosis in U-87 cells and the combination of two drugs further significantly enhanced the apoptosis ratio. NAC partially protected cells from the apoptosis induced by the drugs.

### 3.4. Effects of Ptx and BA on the Intracellular ROS Generation in U-87 Cells

Fluorescence microscopy with H_2_DCF-DA as a fluorescent probe was performed to estimate the intracellular ROS accumulation in U-87 cells. As shown in Figure 4, H_2_O_2_, as a positive control, induced the most dramatic increase of ROS levels. There was also an obvious increase in intracellular ROS production after U-87 cells stimulated with BA, whereas the combination of Ptx and BA resulted in a statistically significant increase in ROS production compared with the effects of either of the drugs alone. However, the induction of intracellular ROS by Ptx/BA combination could be partly alleviated by NAC pretreatment, which paralleled the effect of NAC on the cytotoxicity of BA and Ptx delivered simultaneously (Figure 4).

### 3.5. Effects of Ptx and BA on Akt Signaling Pathway in U-87 Cells

**Figure 5:**
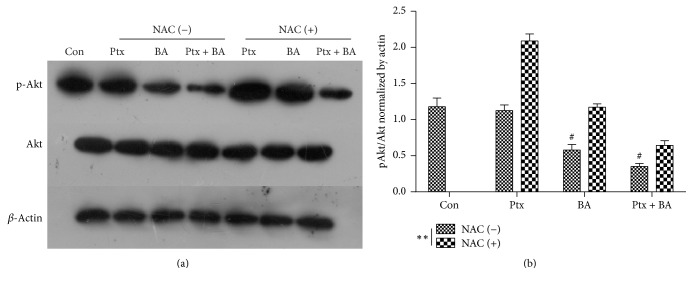
Protein expression of Akt and p-Akt in U-87 cells exposed to 1.5 *μ*g/mL BA and 1 ng/mL Ptx in single or in combination with or without the pretreatment of NAC. (a) Blot image of the protein expression. (b) Bar graph representing the semiquantification of gel image (p-Akt/Akt) normalizing the band with the actin control. *∗* means *p* < 0.05 versus the corresponding group. # represents *p* < 0.05 versus control. Data are presented as mean ± SD (*n* = 3). *∗∗* refers to *p* < 0.01 versus the corresponding group.

It is showed in Figure 5 that BA and Ptx, singly or in combination, could inhibit the activation of Akt pathway by repressing the expression of p-Akt. Combinational exposure to BA and Ptx led to more decrease of p-Akt. In addition, NAC pretreatment partially reversed the inhibitory effects of either single or combinational administration of Ptx and BA on the expression of p-Akt. Quantitative analysis indicated that combinational application of Ptx and BA showed significantly stronger effect to inhibit the expression of p-Akt (*p* < 0.05). Moreover, NAC pretreatment significantly counteracted the inhibitory effect of either drug in single or combination on the expression of p-Akt (*p* < 0.05) (Figure 5).

### 3.6. In Vivo Antitumor Effect of BA and Ptx

**Figure 6:**
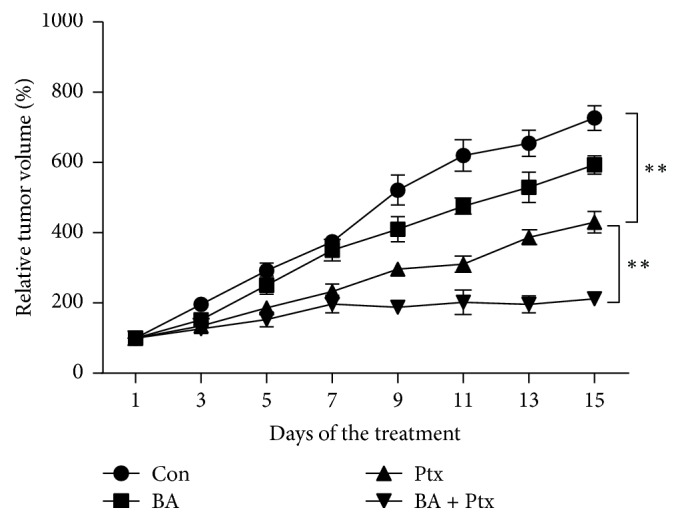
In vivo evaluation of either single or combinational treatment of BA and Ptx. *∗∗* means *p* < 0.05 versus the corresponding group.

Figure 6 showed the tumor volume of the U87 xenograft model when treated with single or combinational administration of BA and Ptx. As shown in Figure 6, BA had a slight antitumor effect while Ptx produced a modest effect of delaying tumor growth. Most importantly, it is noted that combinational delivery of both BA and Ptx generated the most significant growth inhibitory effect. For example, at the end of the experiment, relative tumor volume from the combinational group was around 200% while that from BA or Ptx group was more than 400% or 600%, respectively.

## 4. Discussion

Most of the previous studies focused on the reversal of mediated multidrug resistance by BA [17]. As reported previously, BA induces cell apoptosis through not only JNK/AP-1 signaling but also targeting Ca^2+^/calmodulin-dependent protein kinase II [12, 16]. In addition, a recent study has demonstrated that BA could synergistically enhance the cytotoxicity of gemcitabine by activating transforming growth factor-*β*/Smad signaling [6]. So far, there is no report about the synergistic antitumor effect of BA and Ptx. Therefore, the molecular mechanism underlying this synergism remains unknown. In the current study, it is reported for the first time that BA could synergistically enhance the cytotoxicity of Ptx by induction of intracellular ROS levels and sequential inhibition of Akt pathway in malignant glioma cells.

As reported in previous study, Ptx chemoresistance correlates very well to intracellular antioxidant capacity. The ratio between cellular antioxidant capacity and reactive oxygen species impacts heavily on the antitumor efficiency of Ptx [16]. Consequently, inhibition of cellular antioxidant capacity such as elevating intracellular ROS levels would probably strengthen the cytotoxicity of Ptx. On the contrary, introduction of antioxidant, such as vitamin E or NAC, could significantly protect tumor cells from apoptosis.

Figure 1 clearly indicates the synergistic cytotoxicity of BA and Ptx against U-87 cells, which is in accordance with the apoptosis rates showed in Figure 3. The mechanism for the synergism lies on the induction of intracellular ROS (Figure 4) and the following suppression of Akt pathway (Figure 5). As demonstrated in previous studies, BA could exert positive influences on the generation of ROS and lead to proapoptotic or antiapoptotic effects [20]. In the current study, simultaneous delivery of BA and Ptx led to significant elevation of ROS than single drug did (Figure 4). The induced effect of intracellular ROS could be reversed to a significant extent by antioxidant ROS scavenger NAC (Figure 4). Meanwhile, MTT assay showed that NAC pretreatment effectively increased the survival of cells exposed to BA plus Ptx (Figure 2). Therefore, the results reported here clearly demonstrate that intracellular ROS induction is necessary for the synergistic cytotoxicity of BA and Ptx.

It has been demonstrated that Akt pathway plays a key role in Ptx chemoresistance [21]. In addition, recent studies reported that regulating the activity of Akt pathway and its downstream apoptotic pathways is of great influence to Ptx resistance in several human cancer cell lines, which featured the central role of Akt involvement in Ptx chemoresistance [22, 23]. Meanwhile, the proapoptotic effect of BA against cancer cells was closely related to the activity of Akt pathway [24, 25]. Therefore, the current study firstly demonstrated that the inhibition of ROS-dependent Akt pathway is essential for the synergistic antitumor efficiency of BA and Ptx. As showed in Figure 5, cells exposed to combined BA and Ptx showed a significantly lower expression level of p-Akt. Pretreatment of NAC could partly abolish this change, which demonstrated that p-Akt is the downstream target of ROS signal. Finally, in vivo evaluation further demonstrated the synergistic effect of the combinational delivery of BA and Ptx with the most significant tumor growth inhibitory effect. Still, intensive study is undergoing in the author's lab to further elucidate the possible mechanism underlying the synergistic effect. Moreover, intracranial model of glioma is to be established to mimic the physiological environment for glioma in human bodies.

In conclusion, the present study reported that BA could synergistically enhance the cytotoxicity of Ptx against U-87 cells not only in in vitro study but also in in vivo evaluation. The possible mechanism may lie on the induction of intracellular ROS and the suppression of Akt pathway by BA. Moreover, it is indicated that intracellular ROS appears to be an upstream regulator of Akt pathway. Therefore, data from this study not only confirm the potential of BA in treating glioma but also offer an effective way to improve the anticancer efficiency of Ptx by combinational delivery of BA.

## Figures and Tables

**Table 1:** IC50 values of Ptx and BA singly or in combination against U-87 cells.

IC50 values	24 h	36 h	48 h
Ptx alone (nM)	199.6 ± 15.4	40.5 ± 3.2	14.2 ± 1.1
Ptx in combination (nM)	1.9 ± 0.2^*∗∗*^	1.6 ± 0.2^*∗∗*^	1.2 ± 0.1^*∗∗*^
BA alone (*μ*M)	30.3 ± 2.3	22.8 ± 1.9	9.8 ± 0.6
BA in combination (*μ*M)	5.4 ± 0.3^*∗∗*^	3.4 ± 0.1^*∗∗*^	2.0 ± 0.2^*∗∗*^

*∗∗* means *p* < 0.01 versus single drug at the same time.
